# Mechanisms and Developmental Roles of Promoter-proximal Pausing of RNA Polymerase II

**DOI:** 10.4172/2157-7633.1000330

**Published:** 2016-03-14

**Authors:** Christine Robinson, Matthew Lowe, Amanda Schwartz, Nobuaki Kikyo

**Affiliations:** Stem Cell Institute, Department of Genetics, Cell Biology and Development, University of Minnesota, USA

**Keywords:** 7SK snRNP, Differentiation, Gene expression, Promoter, P-TEFb, RNA polymerase II, Stem cells

## Abstract

RNA polymerase II (Pol II) temporarily stops transcription after synthesizing 30–50 bases, and resumes elongation only after stimulations by various signaling molecules and developmental cues. This phenomenon, called promoter-proximal pausing, is observed in 10–50% of the entire genes from *Drosophila* embryos to human cells. Release of paused Pol II is primarily mediated by the activated form of positive transcription elongation factor b (P-TEFb) initially sequestered in the inhibitory 7SK small nuclear ribonucleoprotein (7SK snRNP) complex. Many proteins and RNAs have been discovered and studied in detail to explain the process of the pausing and release of Pol II in relation to P-TEFb. At the functional level, promoter-proximal pausing regulates genes involved in stimulus-response and development in *Drosophila*. In mammalian stem cell biology, pausing is important for proliferation and signaling in embryonic stem cells and the formation of induced pluripotent stem cells. Other than this, however, little is known about the biological significance of pausing in mammalian cell differentiation. Further study on pausing mechanisms as well as its functions will contribute to the development of stem cell biology and its clinical applications.

## Introduction

**Figure 1:**
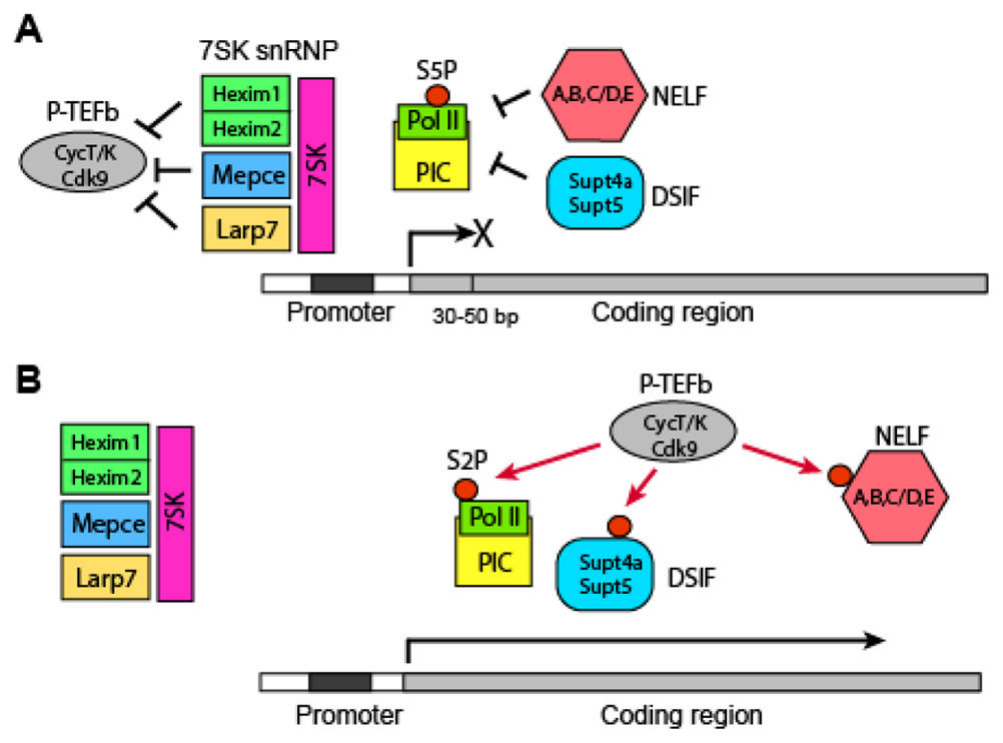
Key players responsible for promoter-proximal pausing of RNA polymerase II. Paused status (A) and released status (B) of RNA polymerase II are depicted.

After RNA polymerase II (Pol II) and general transcription factors are assembled to form the pre-initiation complex (PIC) at a promoter, it synthesizes a short mRNA of 30–50 bases and then pauses (promoter-proximal pausing or PPP) [1–6] (Figure 1A). PPP is induced by two mechanisms. First, the PIC is directly inhibited by two protein complexes: 5,6-Dichloro-1-β-D-ribofuranosylbenzimidazole (DRB) sensitivity-inducing factor (DSIF, containing Supt4a and Supt5) and negative elongation factor (NELF, composed of subunits A-E). Second, the promoter of transcription elongation, positive transcription elongation factor b (P-TEFb, comprised of Cdk9 and either CycT1, CycT2, or CycK), is sequestered in the inhibitory complex 7SK small nuclear ribonucleoprotein (7SK snRNP). In 7SK snRNP, the noncoding RNA 7SK serves as a scaffold to assemble four protein subunits (Hexim1, Hexim2, Larp7, and Mepce) around P-TEFb [7]. Various proteins and RNAs release P-TEFb from 7SK snRNP, activating the Cdk9 kinase. As a result, Cdk9 releases NELF and converts DSIF into an elongation-promoting factor, both via phosphorylation (Figure 1B). Cdk9 also phosphorylates serine 2 (S2P) on the carboxy-terminal heptapeptide repeat domain (CTD) in the largest subunit of Pol II, facilitating the recruitment of RNA-processing machinery and chromatin remodeling factors. In contrast, paused Pol II is characterized by the phosphorylation of serine at the 5^th^ position (S5P) on CTD. PPP is highly prevalent; between 10% and 50% of entire genes simultaneously undergo PPP in many cell types, with only around a half of them proceeding to transcription elongation [1,8–10]. As expected from this prevalence, Pol II is released from PPP through many mechanisms in different contexts. This review article will first describe experimental approaches commonly used to study PPP, and then proceed to the mechanisms underlying the release of P-TEFb from 7SK snRNP and the recruitment of P-TEFb to its target genes, two rate-limiting steps towards the release of Pol II from PPP. Finally, we will discuss the regulation of PPP during *Drosophila* development and pluripotent stem cell differentiation, as they are the most widely studied models.

### Genome-wide approaches to examine PPP

By definition, the study of PPP requires the detection of the distribution pattern of Pol II within a given gene, which is commonly performed with chromatin immunoprecipitation (ChIP) followed by a microarray analysis (ChIP-chip) or next generation sequencing (ChIP-seq) (Table 1). These approaches have the strength of detecting not only Pol II and its phosphorylated forms but also several covalent histone modifications characteristic of paused genes, all within the same set of experiments. ChIP-chip studies have demonstrated that the promoters of paused genes are typically enriched with histone marks characteristic of initiated genes, such as trimethylated lysine 4 of histone H3 (H3K4me3) and acetylated H3K9 and H3K14, in addition to Pol II S5P [8]. Additionally, paused genes usually lack markers of transcriptional elongation at the coding regions, including H3K36me3, H3K79me2, and Pol II S2P.

Definitive evidence for paused transcription requires detection of transcript length. Global Run-On Sequencing (GRO-seq) is a genome-wide nuclear run-on assay, in which nascent transcripts are labeled with 5-bromouridine 5′-triphosphate (BrUTP) [9]. The labeled RNAs are then purified using anti-deoxy-BrU beads and sent for next generation sequencing. This approach is particularly useful in determining how specific stimuli release paused Pol II, allowing for elongation at a specific time point. Precision Run-On Sequencing (PRO-seq) sequencing is a modification of GRO-seq, in which reactions incorporate biotinylated NTPs. Incorporation of the first biotinylated NTP inhibits further elongation, allowing for the detection of nascent transcripts at a single-base resolution, as opposed to 30 to 50-base resolution with GRO-seq [11].

Capping of mRNA with 7-methylguanosine takes place at the 5′ end during the initial stage of transcription [12]. The cap protects mRNA from exonuclease degradation, allowing for transcription to complete. Therefore, short mRNAs (<100 bases) with a 5′ cap can be isolated and sequenced to identify paused mRNAs using a technique known as short-capped RNA analysis [13].

Among these genome-wide approaches, ChIP-based approaches are more readily available to general cell biology laboratories than mRNA-based approaches, which require extensive optimization. A recent report showed that GRO-seq data are generally consistent with ChIP-seq data obtained using anti-Pol II antibody [14]; current PPP research typically employs the combination between GRO-seq and ChIP-seq of Pol II.

### Release of P-TEFb from 7SK snRNP

About half of P-TEFb in the nucleus is sequestered in 7SK snRNP [15]. The release of P-TEFb from 7SK snRNP represents one of the first steps toward the release Pol II from PPP. A diverse range of factors, including binding proteins, phosphatases, proteases, and RNAs, are involved in this step (Table 2) and are not mutually exclusive. Several representative examples are highlighted below.

Exposure of cells to ultraviolet light or hexamethylene bisacetamide (HMBA) disrupts 7SK snRNP and releases P-TEFb [16]. Both agents activate calcium ion-calmodulin-protein phosphatase 2B (PP2B), which relaxes the structure of 7SK snRNP although the details remain to be studied. The relaxation allows the second phosphatase, protein phosphatase 1α (PP1α), to gain access to the exposed Cdk9, dephosphorylating threonine at the 186^th^ position (Thr186) in the T-loop of Cdk9. This, in turn, leads to the release of P-TEFb from 7SK snRNP. The dephosphorylation of Thr186 is induced in a physiological context as well. For example, nuclear factor-κB (NF-κB) and the human immunodeficiency virus (HIV)-1 protein Tat recruit PPM1G (protein phosphatase, Mg^2+^/Mn^2+^ dependent, 1G) to promoters, which also dephosphorylates Thr186 [17]. However, the phosphatase-mediated process may not be the only mechanism Tat uses to release P-TEFb from 7SK snRNP. This is due to its ability to bind and release P-TEFb from 7SK snRNP without the use of other factors *in vitro* [18]. Rather, the *in vitro* process is accompanied by a conformational change of 7SK RNA and the release of Hexim1 from 7SK RNP.

The human I-mfa domain containing protein (HIC) interacts with CycT1 to inhibit P-TEFb-dependent transcription [19]. In contrast, HIC mRNA has the opposite effect on transcription. The 3′ untranslated region (UTR) of HIC mRNA is necessary and sufficient to activate gene expression at the HIV-1 promoter by displacing 7SK RNA from P-TEFb, allowing it to dissociate from 7SK snRNP [20]. This is explained by a competition between the similar hairpin structures at the 3′ end regions of HIC mRNA and 7SK RNA. The opposite functions of HIC protein and HIC mRNA illuminate the complexity of P-TEFb release from 7SK snRNP and the many facets of pause regulation in transcription.

Release of P-TEFb is also directly linked with splicing of nascent mRNA. Serine/arginine-rich splicing factor 1 and 2 (SRSF1 and SRSF2), major components of the splicing machinery, bind 7SK snRNP at active gene promoters [21]. When an exonic-splicing enhancer sequence in nascent mRNA, such as GAAGGA, binds SRSFs, they are released from 7SK snRNP along with P-TEFb, allowing for pausing release. Due to the wide distribution of SRSFs in the genome, this mechanism is likely to be used in many contexts of pause release.

All of the previously described release mechanisms are reversible; however, an irreversible mechanism also exists. During megakaryocyte diferentiation, a calcium influx activates the protease calpain 2, causing the cleavage of the Mepce subunit of 7SK snRNP, promoting the release of P-TEFb and upregulating cytoskeleton remodeling genes [22]. GATA1 is known as an upstream transcription factor regulating calpain 2 expression. A truncated mutant GATA1 in megakaryocytic leukemia fails to upregulate calpain 2, revealing the connection between Pol II release, cell differentiation, and leukemogenesis.

### Recruitment of P-TEFb

P-TEFb released from 7SK RNP must to be recruited to the promoters of target genes. This recruitment process is also diverse (Table 3), frequently using multiple mechanisms within a single recruitment, as exemplified by Brd4 and Mediator summarized below. Recruitment of P-TEFb by c-Myc and MyoD will also be discussed as examples directly relevant to cell differentiation. Note that the release of P-TEFb from 7SK snRNP and the recruitment of the released P-TEFb to promoters are not necessarily sequential steps. These processes are not chronologically indistinguishable in some cases because a single protein, like Brd4, can be involved in the both steps.

Brd4 is a member of the bromodomain and extraterminal domain (BET) family proteins [15,23]. Brd4 is ubiquitously expressed and binds free P-TEFb that is released from 7SK snRNP. Because Brd4 binds acetylated histone H3 and H4 via bromodomains, it was initially proposed to recruit P-TEFb to acetylated promoters. However, it was later found that bromodomains are not required for Brd4 to recruit P-TEFb to promoters [24]. Instead, Brd4 is recruited to promoters by Mediator or other chromatin components. Additionally, Brd4 interacts with jumonji C-domain-containing protein 6 (JMJD6) at enhancers and regulates Pol II release from PPP through long-range interactions between enhancers and promoters [25]. In this context, dual demethylase activity of JMJD6 plays a critical role in transcription elongation. First, protein demethylase activity of JMJD6 is directed toward symmetric dimethylation of H4R3 (H4R3me2s), a marker for gene suppression, and contributes to gene activation. Second, RNA demethylase activity of JMJD6 removes the methyl group at the 5′ cap in 7SK RNA (decapping), which destabilizes 7SK snRNP, resulting in the release of P-TEFb. In human embryonic kidney cells HEK293T, more than 1,200 genes are co-regulated by Brd4 and JMJD6, suggesting the prevalence of this mechanism.

P-TEFb is also recruited to promoters by Mediator through multiple mechanisms. Mediator is a protein complex composed of 26 core subunits serving as a physical and functional bridge between Pol II and DNA-bound transcription factors during transcription initiation and elongation [26,27]. The MED26 subunit recruits P-TEFb as a part of the super-elongation complex (SEC) to the promoters of *c-Myc* and *hsp70* [28]. The recruitment of SEC is particularly important because it contains other subunits that promote elongation after pause release, such as the eleven-nineteen lysine-rich in leukemia (ELL) family members and ELL-associated factors (EAFs) 1 and 2. Additionally, the interaction between Mediator and SEC is induced by hypoxia-inducible factor 1A (HIF1A) in hypoxic breast cancer cells [29]. Mediator also uses another subunit MED23 to recruit P-TEFb to the promoter of the serum-responsive gene *Egr1* in mouse embryonic stem cells (ESCs) through a direct interaction with Cdk9 [30].

The oncoprotein c-Myc directly binds Cdk9 and CycT1 at different domains and recruits them to target genes [31,32]. In mouse ESCs, c-Myc binds a third of actively transcribed genes [33]. Treatment of ESCs with a chemical inhibitor of c-Myc or knockdown of c-Myc with shRNA reduces Pol II in the coding regions of the target genes without affecting the level of Pol II at promoters. This provides evidence that c-Myc is responsible for regulating the release of Pol II from pausing. Because c-Myc is one of four genes used to establish induced pluripotent stem cells (iPSCs) [34], its release of Pol II from paused genes may be one of its functions during iPSC formation.

MyoD is a master regulator of muscle differentiation [35]. It interacts with P-TEFb in the proliferating and early differentiation phases of myoblasts [36]. The interaction in the early phase of differentiation brings P-TEFb to MyoD target genes specific to differentiation, such as myogenin and muscle creatine kinase [37]. The functions of the interaction in the proliferating phase remains unclear; however, the ability of Cdk9 to phosphorylate MyoD may regulate its activity independently of pausing release [36].

### Developmental roles of PPP in *Drosophila*

PPP was discovered in 1986 at the heat shock gene *hsp70* in a *Drosophila* cell line [38]. However, it took more than 20 years before genome-wide prevalence of PPP – 10–20% of all *Drosophila* genes – was discovered in 2007 by applying the ChIP-chip technique [39,40]. While a large number of paused genes are related to heat shock and other stimulus-responses, nearly a third of the paused genes play roles in development, such as cell differentiation and cell-cell communication [39]. These results led to the interpretation that PPP acts as a checkpoint to control precisely-timed gene activation during development. This notion is consistent with the initial discovery of PPP at *hsp70*, which is rapidly activated upon a heat stimulus.

Paused developmental genes include those involved in embryonic axis formation and gastrulation in *Drosophila*. The bone morphogenetic protein (BMP) pathway regulates dorsal-ventral patterning and 25 genes involved in this pathway are paused [41]. The segmentation genes (gap, pair-rule, and segmentation polarity genes), which specify the anterior-posterior axis, are also paused. Among many paused developmental genes, timely release of Pol II is particularly important for the *Snail* gene (*sna*), which is a key regulator for epithelial-mesenchymal transition and mesoderm formation [42,43]. *sna* is expressed in around 1,000 presumptive mesoderm cells and synchronized activation of the gene is essential for coordinated invagination of the cells. When the tightly paused *sna* promoter was swapped with the moderately paused *sog* gene promoter, it resulted in a mixed embryonic population with normal and partial invagination. Replacement with the non-paused *ths* promoter completely abolished invagination in the majority of embryos, and normal invagination was rarely detectable.

Studies with *Drosophila* also provided an unexpected insight into a novel mechanism of PPP. Knockdown of NELF was supposed to primarily upregulate gene expression by releasing Pol II; however, it upregulated 71 genes and downregulated 170 genes out of 18,500 genes in total [44]. The downregulated gene promoters displayed not only decreased Pol II, as expected by the lack of NELF, but also increased nucleosome occupancy, a potential mechanism for the observed downregulation [44,45]. This finding suggested that Pol II and nucleosomes compete against each other for occupancy at the promoter regions in the downregulated genes. Because NELF knockdown also increased many genes, there must be additional regulatory mechanisms that dictate the final outcome of the gene expression. In addition, a recent report showed that pausing is not a simple on/off state; 90% of transcriptionally active genes (7,036 out of 7,734) are partially paused in *Drosophila* embryos [41]. These findings indicate the presence of multiple layers of fine tuning mechanisms that await further study.

### Roles of PPP in pluripotent stem cells

Pluripotent stem cells and hematopoietic cells have been the main cell types used for the study of the developmental role of PPP in mammalian cells. The focus of this section will primarily be upon pluripotent stem cells due to the more in depth mechanistic and genome-wide studies compared to hematopoietic cells [22,46–48]. In fact, the first genome-wide distribution of PPP in mammalian cells was described in human ESCs [8]. The occurrence of PPP in ESCs has been analyzed using GRO-seq and ChIP-seq of Pol II in mouse ESCs as well as ChIP-chip of Pol II in human ESCs (Table 4).

In contrast to the initial findings in *Drosophila* of a direct link between PPP and the regulation of development, there appears to be no definitive link in mammalian cells. The number of RefSeq genes paused in mouse ESCs and MEFs, 39% and 34% respectively, is quite similar [10]. In both cell types, paused genes are primarily from the cell cycle, catabolism, and DNA damage response categories as shown by a gene ontology analysis of the GRO-seq data. Pol II was not detected at the pluripotency genes in MEFs, consistent with the lack of expression. Unexpectedly, the pluripotency genes of ESCs show significant enrichment of Pol II within the promoters compared to the coding regions, suggesting that at least partial PPP does occur at these loci. The meaning behind the partial PPP requires further study to elucidate its role in the maintenance of pluripotency. The prevalence of PPP in human cells is also similar between pluripotent cells and differentiated cells as observed with ChIP-chip of Pol II-S2P in hepatocytes (36% of protein-coding genes), B cells (42%), and ESCs (52%) [8]. The categories of paused genes have yet to be extensively characterized in human pluripotent stem cells.

Mouse ESCs have traditionally been cultured in the presence of serum; however, a serum-free condition called the 2i system, which uses two kinase inhibitors, has seen widespread use as a more chemically-defined culture system recently [49]. After being cultured with serum, ESCs display leaky expression of many lineage-specific genes and heterogeneous expression of pluripotency genes [50]. Unexpectedly, cells cultured under the 2i condition demonstrate lower expression of lineage-specific genes as well as a more homogenous expression of pluripotency genes than the cells grown in serum-based media [49,51]. This is not due to a lack of PPP in ESCs cultured in the presence of serum. In a comparative GRO-seq analysis of PPP in the cells cultured with serum and the 2i, there was no significant difference in the gene families that were paused between the two conditions [14]. The genes that showed the highest levels of PPP in both culture conditions were those involved in signal transduction and the regulation of cell cycle, not differentiation or pluripotency. This suggests that PPP does not play a significant role in the regulation of developmental genes in ESCs.

In a more functional sense, the manipulation of PPP during reprogramming of MEFs to iPSCs can provide a new approach to improve the quality and efficiency of cell reprogramming. During reprogramming there are two waves of gene activation, the first being genes related to proliferation, metabolism, and cytoskeleton whereas the second is primarily the pluripotency genes and their regulators [52]. During reprogramming there is significant PPP in 55% of the second wave genes, most notably Oct4 [52]. The role of PPP in reprogramming was further studied by knocking down several key genes involved in the sequestration and release of P-TEFb. The elimination of PPP via downregulation of Hexim1 or upregulation of Brd4 accelerates the formation of iPSCs as well as increasing the number of iPSC colonies [52]. Alternatively, the upregulation of Hexim1 or downregulation of Brd4 leads to an arrest in reprogramming prior to the second wave. Beyond the factors directly involved in the PPP machinery, the pluripotency factor Klf4 recruits Cdk9 to pluripotency genes during reprogramming as well as in ESCs [52]. Knockdown of Klf4 leads to a significant decrease of Cdk9 loading on the pluripotency genes and their expression. Additionally, a majority of the genes involved in the late stage of reprogramming are targets of Klf4, suggesting a major role for PPP release in the formation of iPSCs. Beyond iPSC formation, by tightly controlling the expression of factors linked to the PPP maintenance and release, it may be possible to more accurately reprogram cells into a wide range of lineages, such as the conversion of fibroblasts to neural cells [53–64].

## Future Directions

Following its discovery, the study PPP has resulted in as many new questions as it has answers. As technology continues to improve, our genome-wide picture of PPP becomes more complex and diverse. The major protein and RNA players have been uncovered; however, our understanding of the mechanisms behind the release and recruitment of those molecules is still incomplete. While the developmental roles for pausing have become more apparent in *Drosophila* and pluripotent stem cells, very little is known about the roles of PPP in the differentiation of many other mammalian lineages. HIF1A-mediated pause release, mentioned above, is evidence that PPP is also involved in cancer and potentially other pathological conditions. In cardiomyocytes, PPP is present at metabolism-related genes, controlling energy homeostasis [54]. These studies help outline the versatility of PPP as a regulatory mechanism whose potential has yet to be understood in its entirety.

## Figures and Tables

**Table 1:** Genome-wide approaches commonly used to detect PPP.

Factor	Principles	References
ChIP-chip and ChIP-seq	Antibodies against Pol II, Pol II S2P, and Pol II S5P as well as various modified histones are used.	[8,14,51,52]
GRO-seq	Nascent RNAs that have incorporated 5-bromouridine 5′-triphosphate are isolated with anti-deoxy bromouridine antibody and sequenced.	[9,10,14]
PRO-seq	Nascent RNAs that have incorporated biotinylated NTP are isolated with streptavidin beads for sequencing	[11]
Short-capped RNA analysis	Short mRNAs (<100 bases) with a 5′ cap are isolated with anti-cap antibody and sequenced.	[12]

Pol II: RNA Polymerase II; Pol II S2P: RNA Polymerase II with phosphorylated serine at the 2^nd^ position in the carboxy-terminal heptapeptide repeat domain; GRO-seq: Global Run-On sequencing; PRO-seq: Precision Run-On sequencing

**Table 2:** Factors associated with the release of P-TEFb from 7SK snRNP.

Factors	Mechanisms	Reference
PP2B and PP1 α	Sequentially dephosphorylate 7SK snRNP and Cdk9	[16]
PPM1G	Recruited by NF-κB and Tat, and dephosphorylates Cdk9	[17]
Tat	Changes confirmation of 7SK RNA	[18]
HIC mRNA	Displaces 7SK RNA from 7SK snRNP by competition	[20]
SRSF1 and 2	Binds the exonic-splicing enhancer sequence in nascent mRNA	[21]
Calpain 2	Cleaves Mepce	[22]
JMJD6	Decaps 7SK RNA and destabilizes 7SK snRNP	[25]
AFF1	Promotes Tat’s extraction of P-TEFb from 7SK snRNP	[55]
PIP7S	Maintains 7SK snRNP integrity	[56]

PP2B: Protein Phosphatase 2B; PP1α: Protein Phosphatase 1 α; 7SK snRNP: 7SK small nuclear ribonucleoprotein; PPM1G: Protein Phosphatase, Mg^2+^/Mn^2+^ dependent, 1G; NF-κB: Nuclear Factor-κB; Tat: Tyrosine aminotransferase; HIC: Human I-mfa domain containing protein; SRSF: Serine/Arginine-Rich Splicing Factor; JMJD6: Jumonji C-Domain-Containing Protein 6; AFF1: AF4/FMR2 Family Member 1; P-TEFb: Positive Transcription Elongation Factor b; PIP7S: P*-*TEFb-Interaction Protein for 7SK Stability

**Table 3:** Factors responsible for the recruitment of P-TEFb to promoters.

Factor	Functions	References
Brd4	Binds free P-TEFb	[15,23]
	Binds Mediator	[24]
	Binds JMJD6 to release P-TEFb	[25]
	Binds Oct4 and regulates pluripotency genes in ESCs	[57]
Mediator (MED26)	Recruits P-TEFb as a part of SEC	[28]
Mediator (MED23)	Binds P-TEFb in ESCs	[30]
HIF1A	Induces the Mediator-SEC interaction	[29]
c-Myc	Directly binds Cdk9 and CycT1	[31,32]
	Binds a third of active genes in ESCs	[33]
MyoD	Binds P-TEFb to activate muscle differentiation genes	[37]
Ikaros	Binds Cdk9 to elongate the γ globin gene in erythroid cells	[46]
GATA1	Binds P-TEFb during megakaryocyte differentiation	[47]
TIF1 γ	Recruits P-TEFb to erythroid genes during erythropoiesis	[48]
Klf4	Recruits P-TEFb during iPSC formation	[52]
NF-κB	Recruits P-TEFb to the IL-8 promoter upon TNFα stimulation	[58]
Cdk8	Binds Mediator and promotes elongation	[59]
STAT3	Binds Cdk9 upon induction by IL-6 in hepatoblastoma cells	[60]
Estrogen receptor α	Overcomes pausing at intron 1 of the MYB gene after binding of estrogen	[61]
Androgen receptor	Binds P-TEFb upon stimulation by androgen in prostatic cancer cells	[62]
PPARγ	Directly binds Cdk9 during adipogenesis	[63]
Aryl hydrocarbon receptor	Recruits P-TEFb to a cytochrome P450 gene upon binding to aryl hydrocarbon	[64]

Brd4: Bromodomain containing 4; P-TEFb: Positive Transcription Elongation Factor b; JMJD6: Jumonji C-Domain-Containing Protein 6; ESC: Embryonic Stem Cell; SEC: Super-Elongation Complex; HIF1A: Hypoxia-Inducible Factor 1A; CycT1: Cyclin T1; GATA1: GATA Binding Protein 1; TIF1γ: Transcriptional Intermediary Factor 1 γ; Klf4: Kruppel-like factor 4; NF-κB: Nuclear Factor-κB; STAT3: Signal Transducer and Activator 3; IL-6: Interleukin 6; PPARγ: Peroxisome Proliferator Activated Receptor γ

**Table 4:** A Comparison of PPP analyses in various cell types.

Cell Types	Methods of detection	Paused percentage	Categories of paused genes	Pluripotency relation	Reference
Mouse ESCs cultured with serum	GRO-seq	39%	Pluripotency, DNA damage response, translation, cell cycle, and catabolism	Pol II is not significantly paused at developmental regulators. Pluripotency genes are partially paused.	[10]
MEFs	GRO-seq	34%	DNA damage response, translation, cell cycle, and catabolism	Not Discussed	[10]
Mouse ESCs cultured with 2i	GRO-seq and ChIP-seq of Pol II	Not discussed	Cell cycle, signal transduction, and cell proliferation	Pol II is not significantly paused at developmental regulators.	[14]
Mouse ESCs cultured with 2i	ChIP-seq of Pol II	Not discussed	Not Discussed	A general increase in PPP after growth in cells cultured with 2i than with serum.	[51]
Mouse iPSCs	ChIP-seq of Pol II	Not discussed	Oct4 in the later stage of reprogramming	Klf4 recruits Cdk9 to the pluripotency promoters.	[52]
Human ESCs cultured with serum	ChIP-chip of Pol II-S5P	52%	Not discussed	Not discussed	[8]
Human hepatocytes	ChIP-chip of Pol II	36%	Not discussed	Not discussed	[8]
Human B cells	ChIP-chip of Pol II	42%	Not discussed	Not discussed	[8]

## Abbreviations

7SK snRNP: 7SK Small Nuclear Ribonucleoprotein
BET: Bromodomain and Extraterminal Domain
BMP: Bone Morphogenetic Protein
Brd4: Bromodomain containing 4
Br-UTP: 5-bromouridine 5′-triphosphate
CTD: Carboxy-Terminal Heptapeptide Repeat Domain
DRB: 5,6-Dichloro-1-β-D-Ribofuranosylbenzimidazole
DSIF: 5,6-Dichloro-1-β-D-Ribofuranosylbenzimidazole Sensitivity-inducing Factor
EAF: ELL-Associated Factor
ELL: Eleven-nineteen Lysine-rich in Leukemia
ESC: Embryonic Stem Cell
GRO-seq: Global Run-On Sequencing
H3K9me3: Trimethylated Lysine 4 of Histone H3
HIC: Human I-mfa Domain Containing Protein
HIF1A: Hypoxia-Inducible Factor 1A
HIV: Human Immunodeficiency Virus
HMBA: Hexamethylene Bisacetamide
JMJD6: Jumonji C-Domain-Containing Protein 6
NF-κB: Nuclear Factor-κB
NELF: Negative Elongation Factor
PIC: Pre-Initiation Complex
PPP: Promoter-Proximal Pausing
Pol II: RNA Polymerase II
PRO-seq: Precision Run-On Sequencing
PP1α: Protein Phosphatase 1α
PP2B: Protein Phosphatase 2B
PPM1G: Protein Phosphatase, Mg^2+^/Mn^2+^ dependent, 1G
P-TEFb: Positive Transcription Elongation Factor b
SEC: Super-Elongation Complex
S2P: Phosphorylated Serine 2
SRSF: Serine/Arginine-Rich Splicing Factor
Thr186: Threonine at the 186^th^ position
UTR: Untranslated Region
